# Identifying spawning sites and other critical habitat in lotic systems using eDNA “snapshots”: A case study using the sea lamprey *Petromyzon marinus *L.

**DOI:** 10.1002/ece3.4777

**Published:** 2018-12-27

**Authors:** Fiona S. A. Bracken, Sean M. Rooney, Mary Kelly‐Quinn, James J. King, Jens Carlsson

**Affiliations:** ^1^ Area 52 Research Group, School of Biology and Environmental Science/Earth Institute University College Dublin Dublin Ireland; ^2^ Inland Fisheries Ireland Dublin Ireland; ^3^ School of Biology and Environmental Science University College Dublin, Science Centre West Belfield Dublin 4 Ireland

**Keywords:** conservation biology, environmental DNA, fish, habitat‐use, lamprey, qPCR, wildlife management

## Abstract

Many aquatic species of conservation concern exist at low densities and are inherently difficult to detect or monitor using conventional methods. However, the introduction of environmental (e)DNA has recently transformed our ability to detect these species and enables effective deployment of limited conservation resources. Identifying areas for breeding, as well as the ecological distribution of species, is vital to the survival or recovery of a conservation species (i.e., areas of critical habitat). In many species, spawning events are associated with a higher relative abundance of DNA released within an aquatic system (i.e., gametes, skin cells etc.), making this the ideal time to monitor these species using eDNA techniques. This study aims to examine whether a “snapshot” eDNA sampling approach (i.e., samples taken at fixed points in chronological time) could reveal areas of critical habitat including spawning sites for our target species *Petromyzon marinus*. We utilized a species‐specific qPCR assay to monitor spatial and temporal patterns in eDNA concentration within two river catchments in Ireland over three consecutive years. We found that eDNA concentration increased at the onset of observed spawning activity and patterns of concentration increased from downstream to upstream over time, suggesting dispersal into the higher reaches as the spawning season progressed. We found *P. marinus *to be present upstream of several potential barriers to migration, sometimes in significant numbers. Our results also show that the addition of a lamprey‐specific fish pass at an “impassable” weir, although assisting in ascent, did not have any significant impact on eDNA concentration upstream after the pass had been installed. eDNA concentration was also found to be significantly correlated with both the number of fish and the number of nests encountered. The application of snapshot sampling techniques for species monitoring therefore has substantial potential for the management of low‐density species in fast‐moving aquatic systems.

## 1 INTRODUCTION

Freshwater biodiversity is facing unprecedented levels of threat and has experienced over 120 extinctions worldwide within the last century (Ricciardi & Rasmussen, 1999). More than 4,600 freshwater species are currently in the threatened or endangered category (IUCN Red List, 2013). Many aquatic species of conservation concern exist at low densities and are inherently difficult to detect or monitor using conventional methods. The introduction of environmental (e)DNA sampling techniques, however, has recently transformed our ability to detect low‐density species and enables more effective and accurate deployment of resources and allocation of time (Ficetola, Miaud, Pompanon, & Taberlet, 2008; Martellini, Payment, & Villemur, 2005; Thomsen et al., 2012). The collection and analysis of eDNA is now becoming commonplace in the detection of freshwater species and assessing biodiversity in aquatic environments (Bohmann et al., 2014; Lodge et al., 2012; Pilliod, Goldberg, Arkle, & Waits, 2013; Taberlet et al., 2012). The probability of detection, however, can vary from species to species and can be dependent on the biology and behavior of the target organism, for example; the amount of DNA they shed, level of activity during sampling period, species density, life cycle stage, and also the type of water body in which they reside (Rees, Maddison, Middleditch, Patmore, & Gough, 2014). Due to the diversity of water bodies and differing quantities of eDNA present in a system, methods for sample collection can vary greatly for rivers or streams, lakes or lagoons, and seawater and are dependent on the size of the environment under study. A range of sampling approaches has previously been employed which have varied in sample size from, for example, *c*. 1,000 × 2 L samples from a canal and waterway system (Jerde, Mahon, Chadderton, & Lodge, 2011), to 5 × 15 ml samples from a sea pen (volume of 4 million liters) within a harbor (Foote et al., 2012). Therefore, the sampling approach can also greatly influence the likelihood of target species detection with an aquatic system.

Quantifying eDNA to estimate the biomass of a target species in running water is invariably complicated and requires the consideration of many variables including eDNA shedding and degradation rate at time of sampling; water temperature; pH; salinity; flow rate; water volume; hydro‐morphology; and the dendritic organization of the habitat (Rees et al., 2014; Roussel, Paillisson, Tréguier, & Petit, 2015; Thomsen et al., 2012). The gathering and utilization of these data are not always possible, or feasible, for the long‐term monitoring of populations. However, studies have shown that within running water systems an increase in the abundance or density of a target species can lead to an increase in either eDNA concentration (Lacoursière‐Roussel, Côté, Leclerc, & Bernatchez, 2016; Pilliod et al., 2013; Takahara et al., 2012; Thomsen et al., 2012) or eDNA detectability (Mahon et al., 2013). Similarly, it has been confirmed that spawning events are characterized by a higher relative abundance of eDNA (Bylemans et al., 2017) making the spawning season an ideal time to utilize eDNA for biomonitoring within lotic systems.

The anadromous sea lamprey (*Petromyzon marinus* L.) was chosen as the target species for this study as their populations are declining across Europe and facing the threat of extinction due to overharvesting, habitat destruction, and the loss of spawning and nursery grounds from the construction of anthropogenic barriers (dams and weirs) blocking upstream access (Almeida, Quintella, & Dias, 2002; Igoe et al., 2004; Kelly & King, 2001; Lucas, Bubb, Jang, Ha, & Masters, 2009; Renaud, 1997). *P. marinus *is anadromous and will migrate back into freshwater to begin their search for suitable spawning grounds. See Maitland (2003) for detailed overview of the life cycle of *P. marinus *and Dawson, Quintella, Almeida, Treble, and Jolley (2015) for details of the larval stage and metamorphosis*.* *P. marinus *spawn on large graveled areas with fast‐flowing water and are thought to identify suitable spawning rivers using pheromones (bile acids) released by larval lampreys residing in the sediment (Li, Sorensen, & Gallaher, 1995; Sorensen & Vrieze, 2003; Sorensen, Vrieze, & Fine, 2003). This increases the chances of finding suitable spawning rivers at the end of their long and costly upriver migration. Lampreys display nest‐building behavior as they reach the spawning grounds, moving large stones and gravel using their oral discs to create a depression in which to spawn (Jang & Lucas, 2005). Typically, within the depression, spawning usually commences with the male attaching to the cephalic/branchial region of the female and wrapping the rest of his body around hers forming a loop. Once the tail loop is tightened, and ready to squeeze the eggs out of the female's body, both male and female will then thrash and vibrate their tails for several seconds, resulting in the expulsion of ova and milt (seminal fluid) into the gravel depression from where it is dispersed downstream with sand and silt particles by water currents (Applegate, 1950). They usually spawn in pairs or groups (i.e., polygamous mating) and will disperse their eggs in nests or shallow depressions in the bed material (Jang & Lucas, 2005) with female *P. marinus *holding up to 114,000–165,000 oocytes (Hardisty, 1970; Hardisty & Huggins, 1970; Maitland, 1980). Spawning may last several days for each female but is dependent on the number of eggs available and numbers of eggs expressed during each spawning act. All lamprey species are semelparous, dying after a single spawning season (Larsen, 1980).

Throughout the spawning season of *P. marinus*, there is consequently a considerable increase in the amount of DNA being released into the environment which is in the form of seminal fluid, ova, sloughed cells from nest building and migratory activity, and necrosing tissue from dead or dying adult lamprey. Yamamoto et al. (2016) determined that eDNA generally provides a “snapshot” of fish distribution and biomass in a large area, and the present study adopted this concept. We employed a strategy to target a low‐density species during the spawning season by taking “snapshots” of the eDNA, that is, a “snapshot” sample is taken at a fixed point in chronological time. This sampling strategy will target a species throughout a period when there is a higher relative abundance of eDNA within a system and will aim to reveal spatio‐temporal trends in eDNA concentration to investigate the distribution of *P. marinus *within our study river catchments. This study aims also to use these snapshots to identify “critical habitat” for our target species, which here is defined as areas of habitat believed to be essential to the species’ conservation (U.S. Endangered Species Act). For the *P. marinus*, critical habitat would specifically include areas used for spawning, as well as habitat utilized during their upstream spawning migration. This approach, however, may be applied in the identification of critical habitat for any aquatic species of interest.

## 2 MATERIALS AND METHODS

### 2.1 Study sites and selection of sampling locations

**Figure 1:**
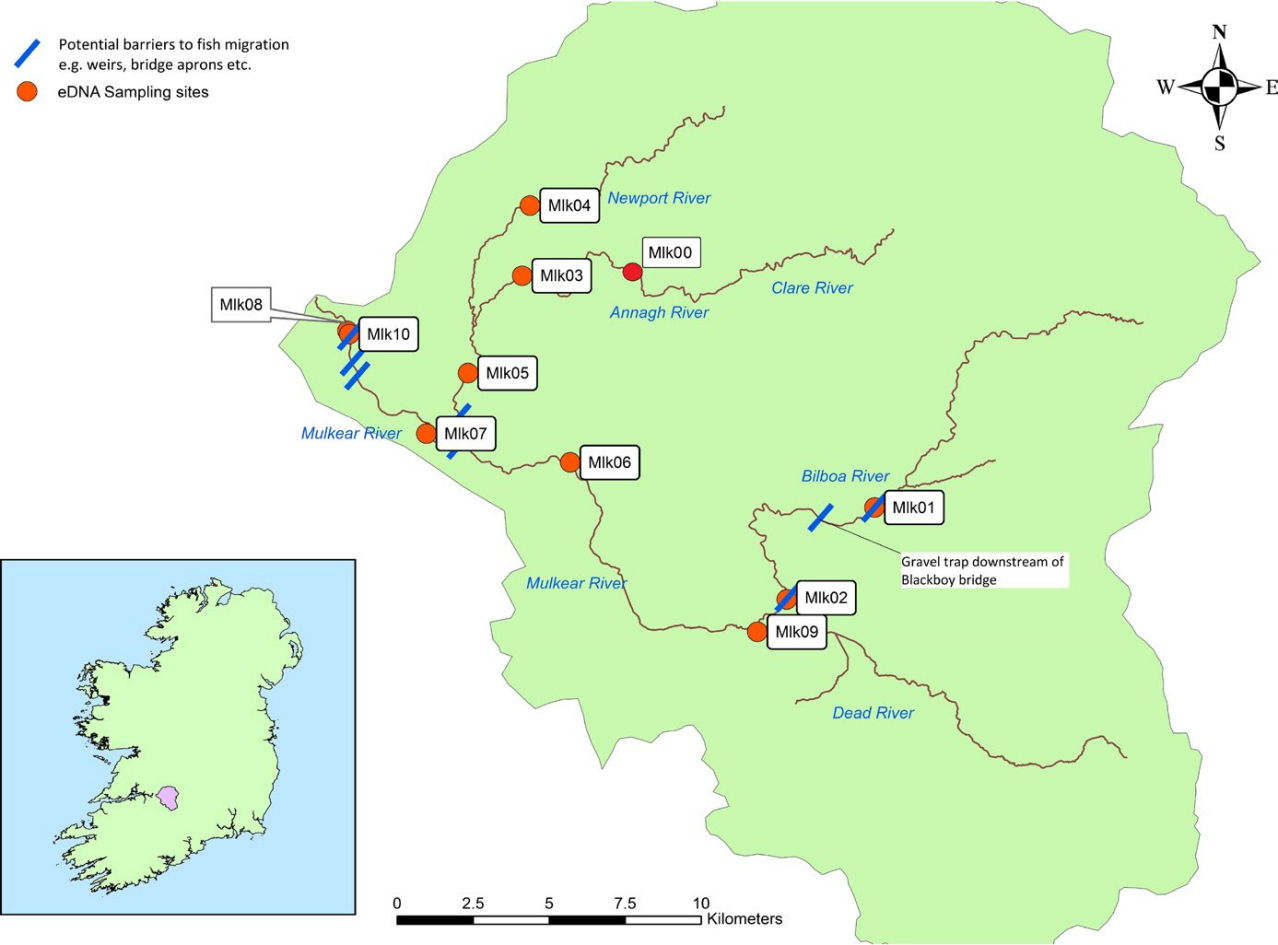
Map of the Mulkear Catchment showing 10 sampling sites and barriers to migration within the catchment. MLK08 is 130 m downstream of MLK10 which is 90 m below Annacotty weir. Note that MLK09 is upstream of the confluence of the Bilboa river and the Dead river

This study spanned a 3‐year period (2015, 2016, 2017) in two separate catchments in Ireland which vary in both spatial scales and in the relative densities of the target species within these catchments (Table 1, Figure 1). “Target Species Density” (Table 1) refers to previous evidence of adult sea lamprey activity within the relevant catchment, in terms of nest counts and/or of individual fish. In excess of 500 sea lamprey and 136 nests were reported below the weir at Annacotty on the Mulkear by Igoe et al. (2004). Likewise, in excess of 50 adult sea lamprey were taken over a single day on the Mulkear by netting for use in a telemetry study by Rooney, Wightman, Ó'Conchúir, and King (2015). In contrast, King and Linnane (2004) had a total nest count of 65 for a float‐over survey using kayaks over a distance in excess of 50 km on the Munster Blackwater (MBW). Based on these figures, we can ascertain that the Mulkear (MLK) has a relatively high density of *P. marinus* as compared to the MBW.

**Table 1:** Physical characteristics of the Mulkear and Munster Blackwater catchments in Ireland

	Catchment area (km^2^)	Main stem length (km)	Target species density	Mean volume discharge (m/s)
Mulkear	650	56	High	12.5
Munster Blackwater	3,324	168	Low	87.5

The Mulkear River has been documented as an important spawning river for *P*. *marinus* (Igoe et al., 2004; Kelly & King, 2001) and forms part of the Lower Shannon Special Area of Conservation (SAC). Ten study sites were chosen within the Mulkear catchment based upon information ascertained from previous spawning surveys using traditional methods (Inland Fisheries Ireland, 2010), and sampling points were positioned at a location downstream of areas where nests have been recorded in the past. MLK10 was introduced to the study as a sampling site in 2016 and is approximately 130 m upstream from MLK08 and 90 m downstream of Annacotty weir. The study sites MLK08 and MLK10 were located just below the Annacotty weir (220 and 90 m respectively) which is 2.2 km upstream from the confluence with the River Shannon (Figure 1). All other sampling sites on the Mulkear river are located upstream of Annacotty weir which poses a potential barrier to *P. marinus *migration (Rooney et al., 2015). However, past assessments have deemed the weir virtually impassable for migrating adult lamprey during flow conditions typical of the spawning season in Ireland (May to July; 3–11 m^3^/s as measured downstream of the weir) (Rooney et al., 2015). In 2016, a lamprey‐specific fish pass was re‐installed at Annacotty weir (prior to the beginning of the lamprey spawning run) which was originally part of the EU Mulkear Life project (Rooney et al., 2015). This gave us the opportunity to also investigate the effect of this lamprey pass on the ability of this species to move upstream. There are smaller weirs also present in the Mulkear catchment (Figure 1) but the dispersal of adult sea lamprey into the catchment points to these features not being a major problem for sea lamprey passage. A gravel trap installed at Blackboys Bridge (Figure 1) is a vertical impediment to upstream fish migration which has a Denil‐type fish pass installed. This trap, as well as the Annacotty weir, was recorded as “impassable” to sea lamprey, in the prevailing conditions at the time of study, in a WFD SNIFFER (2010) fish passage assessment (Barry, Coghlan, Cullagh, Kerr, & King, 2018). The Annacotty weir is the most significant structure that may impede sea lamprey passage in this system, however, within 2 km upstream of this structure, migrants also encounter a crump weir fish counter and the remnants of a weir breached to permit sediment transport and fish passage. There is also a low‐level gauging station crump weir structure at MLK02 and a sloped bridge apron at MLK01 which pose potential barriers also.

**Figure 2:**
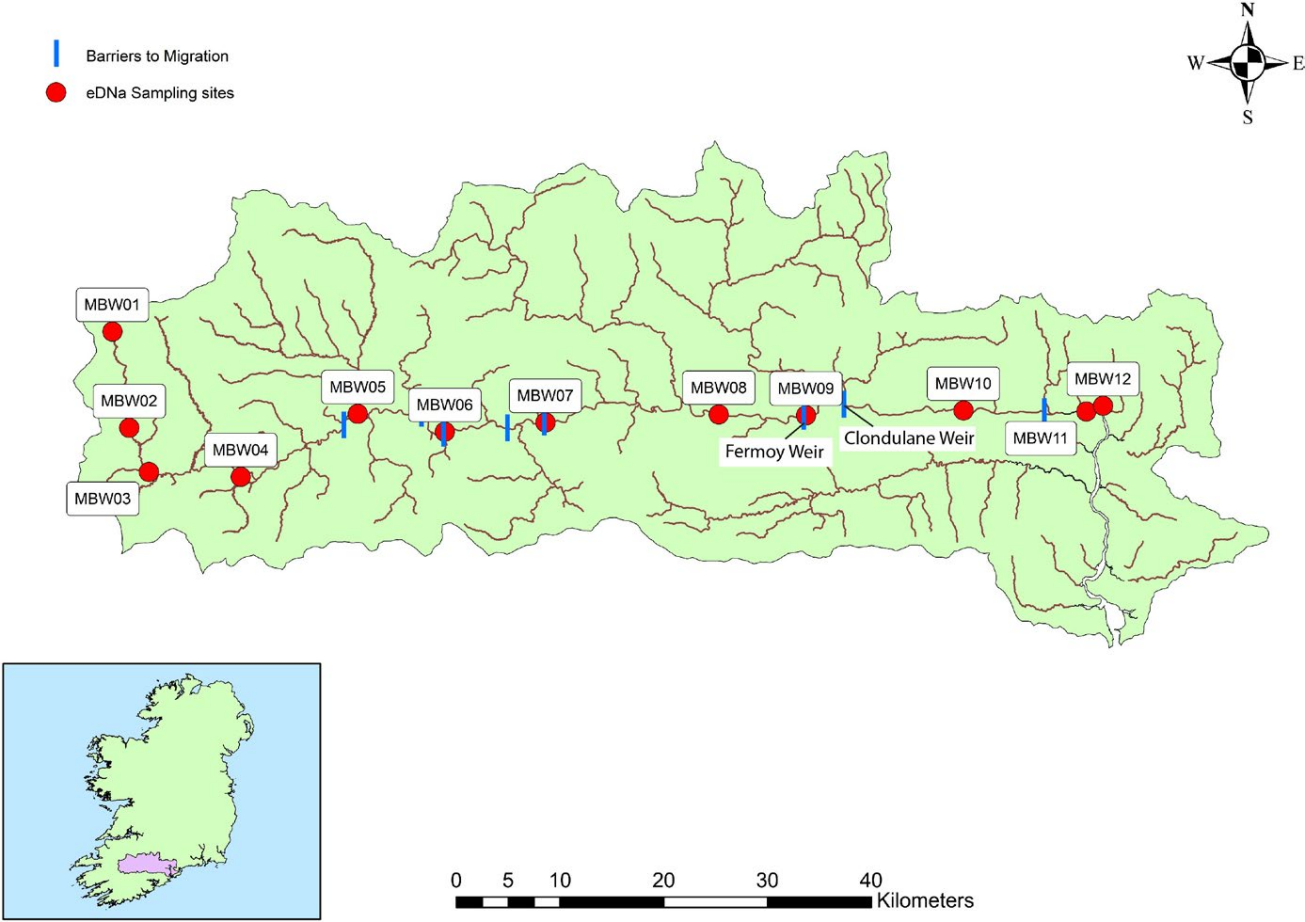
Map of the Munster Blackwater Catchment (MBW) in southern Ireland showing the location of all 12 sampling sites along the main stem of the river as well as barriers to migration. Note that MBW11 and MBW 12 are at the tidal limit

The MBW is relatively a much larger catchment than the Mulkear with a substantially larger mean volume discharge (Table 1). It is one of Ireland's largest and longest river systems and is also a designated SAC for *P. marinus*. Twelve sampling sites were designated along the main stem of the river based on ease of access and coverage of system from source to the tidal areas. Two major weirs are present in the lower part of the main stem. Clondulane Weir is located 25 km upstream of the tidal limit (between MBW09 and MBW10; Figure 2) with a further weir in the town of Fermoy, 4 km further upstream (Figure 2). Both are intact, full‐channel width structures with hydraulic head difference of 2.5 m. Both structures were assessed as “Impassable” to sea lamprey in the prevailing conditions, using the SNIFFER (2010) or WFD 111 protocol (Barry et al., 2018). Evidence from previous spawning surveys has indicated significant *P. marinus* spawning effort downstream of both structures (Inland Fisheries Ireland, 2010; King & Linnane, 2004) and has shown that some degree of passage upstream of the first weir (Clondulane) can occur in years with higher flows (King & Linnane, 2004; IFI, unpublished data). However, the two weirs combined appear to impede further upstream passage for sea lamprey almost entirely. Additional potential barriers in sequence, upstream of these structures include three bridge locations (MBW 07—Mallow town; Lombardstown Bridge upstream of Mallow; Roskeen Bridge MBW 06) where sills or aprons can create very shallow water on the bridge floor and a low vertical drop from the bridge floor. A third weir is located upstream of MBW 05 and downstream of MBW 04.

### 2.2 Field survey

From late May to early June, increases in water temperature above 15°C correspond with the commencement of *P. marinus* spawning activity within areas of suitable habitat (Kelly & King, 2001). Site visits were conducted from early May onwards each year, and once spawning activity was visually confirmed (by either the presence of a nest or a sea lamprey nest building) within one of the study catchments, eDNA sampling commenced. Samples from all sites within one catchment were collected over the course of 1 day (i.e., either all sites on the Mulkear or all sites on the MBW were sampled). Snapshot eDNA samples (1 L water pooled from the left, center, and right sides of river (~333 ml from each) in a sterile bottle) were taken from each sampling site on a series of occasions roughly a week apart (sampling only at lower flows that were safely wadeable) throughout the duration of the spawning season. In the Mulkear, snapshot sampling occurred on six dates throughout the spawning season in 2015, five dates in 2016, and on two dates in the spawning season in 2017. In 2017, replicate samples (2 × 1 L) were taken in tandem in the Mulkear to examine the disparity between replicate samples. In the MBW catchment, sampling occurred on two sampling dates each year (2015, 2016, 2017). A cooler blank was included on each sampling date (the cooler blank contained 1 L deionized water, which was brought to the field, and was treated identically to the other water sampling bottles except that it was not opened at the field sites), as was a field blank (a 1 L water sample which should not contain any *P. marinus *DNA) which was taken at a site upstream of an impassable waterfall in the Mulkear (MLK00; Figure 1) and at the source of the Blackwater (MBW01; Figure 2) where no *P. marinus* should be present. Water samples were immediately stored in an insulated cooler box filled with ice for transport and stored overnight at 4°C until laboratory‐based filtration the following day.

In conjunction with each water sampling event, spawning counts (individual and nest counts) were also conducted. In the Mulkear, this was carried out below Annacotty weir within a 230 m area from the weir to just below sampling site MLK08. In the MBW, counts were made downstream of Fermoy Weir (MBW09; from the weir 100 m downstream). Spawning counts were conducted in the same manner as lamprey surveys are traditionally carried out by wading through the area (after water samples had been taken), counting nests and counting all lamprey that were visible. Spawning counts conducted as such will inevitably fail to account for all individuals present in the area due to them being hidden under banks, rocks, or within deep pools. However, counts do provide a good proxy for spawning activity as well as an indication of the advancement of the spawning season, for example, no new nests/compound nests indicate the season is coming to an end.

### 2.3 DNA extraction and filtration

All laboratory work was conducted in a dedicated eDNA laboratory where DNA extractions and PCR procedures were conducted in two separate laminar flow hoods with a UV light to avoid cross contamination of samples. Each water sample was filtered through individual 0⋅45 μm Whatman cellulose nitrate filters within 24 hr of collection. The filters were dehydrated with 100% ethanol before storage at −20°C, in 2015, but in 2016 and 2017 were frozen directly at −20°C without ethanol. Each filter was subsequently cut into half (half for analysis and half for archival storage) and extracted using Chelex^®^ Chelating resin using a modified protocol from Estoup et al. (1996). Briefly, filters were cut into small pieces within a 2‐ml tube using some fine forceps. A volume of 500 µl of Chelex^®^ (10%) and 20 µl proteinase K (0.1 mg/ml) was added to the tubes and left to digest at 56°C for 2 hr whilst shaking. The temperature was then increased to 99°C for a total of 15 min and left to cool before centrifugation (6,000 ***g*** for 10 min). The supernatant was then transferred to a separate tube and stored at −20°C until use in qPCR analysis.

### 2.4 qPCR Amplification and eDNA quantification

Concentrations of eDNA in samples were determined by qPCR using an Applied Biosystems ViiA 7 (Life Technologies, Inc., Applied Biosystems) quantitative thermocycler in combination with a species‐specific *P. marinus* and *Salmo trutta* assay (Gustavson et al., 2015). Respectively, the sequences for *P. marinus *and *S. trutta *primers (*PmaForward*: 5′‐TTGGAGGCTTTGGCAACTG‐3′ and *PmaReverse*: 5’‐TGTTTATACGAGGGAAGGCCATA‐3′, *StrFoward*: 5′‐TTTTGTTTGGGCCGTGTTAGT‐3′ and *StrR*: 5′‐TGCTAAAACAGGGAGGGAGAGT‐3′) and 5′‐6‐FAM‐labeled minor groove‐binding probes (*Pma*: 5′‐CTAATACTTGGTGCTCCTG‐3′ and *Str*: 5′‐ACCGCCGTCCTCT‐3′) were used which targeted a locus within the mitochondrial cytochrome oxidase I (*coI*) region.* S. trutta* was used as a positive control to ensure that amplifiable DNA was present in the samples as this species is present in abundance in both the Mulkear and the MBW catchments. Amplification reactions included: 15 μl of TaqMan Environmental Master Mix 2⋅0 (Life Technologies, Inc., Applied Biosystems), primers (final concentration of 0⋅2 μM), probe (final concentration of 0⋅2 μM), double‐distilled H_2_O and DNA template (3 μl), forming the 30 μl reaction volume. The qPCR cycling condition was as follows: 50^∘^C for 5 min and 95^∘^C for 10 min, followed by 40 cycles between 95^∘^C for 15 s and 60^∘^C for 1 min. Standard curves for *P*. *marinus *(starting concentration 64.5 ng/μl using seven 10:1 serial dilutions) were generated using DNA extracted from tissue and quantified using fluorometric quantitation (Qubit, ThermoFisher). All samples were quantified in triplicate (technical replicates) with three laboratory negative controls and *P*. *marinus *standard curves as positive controls. *C*q values beyond the dynamic range (i.e., below 6.45 × 10^−^^6^ ng/μl) were interpreted as concentrations of eDNA that were effectively zero.  Here, we use the quantification cycle (Cq), as opposed to the threshold cycle (Ct), to describe the fractional PCR cycle used for quantification in accordance with Bustin et al. (2009).

### 2.5 Data analysis

Data collected from nest counts and individual *P. marinus* counts at MLK08 and MLK10 (combined for correlation analysis due to proximity of sites) were compared to eDNA samples taken on the same dates. First differencing (a transformation method i.e., performed by subtracting the previous observation from the current observation) was used to remove time‐series auto‐correlation. A Pearson's correlation test was carried out in IBM SPSS statistics (v.24) to examine this relationship. ArcMap (v10.4) was used to spatially visualize the temporal patterns in eDNA concentration. Wilcoxon signed‐rank tests (SPSS) were used to compare the eDNA concentration at each sampling site, before and after the addition of a lamprey pass at Annacotty weir.

## 3 RESULTS

### 3.1 “Snapshots” provide an overview of the spatial distribution of the target species within a catchment to reveal spawning aggregations and critical habitat

**Figure 3:**
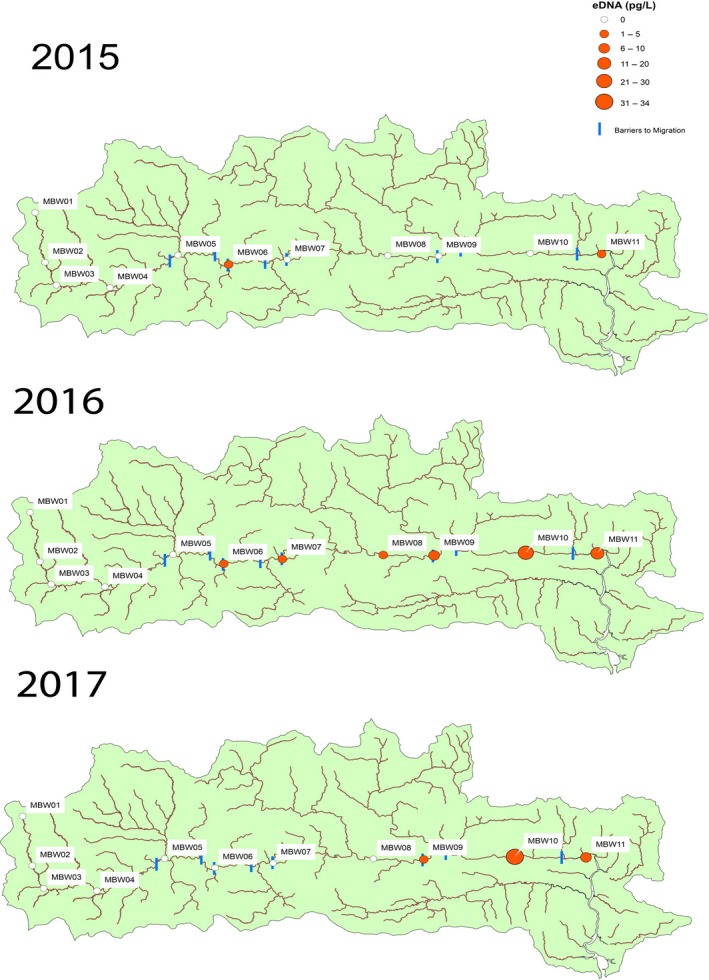
Total eDNA (pg/L) collected at each sampling site within the Munster Blackwater (MBW) catchment. Combined total amount of eDNA collected on each sampling occasion from each year, respectively, 2015–2017

**Figure 4:**
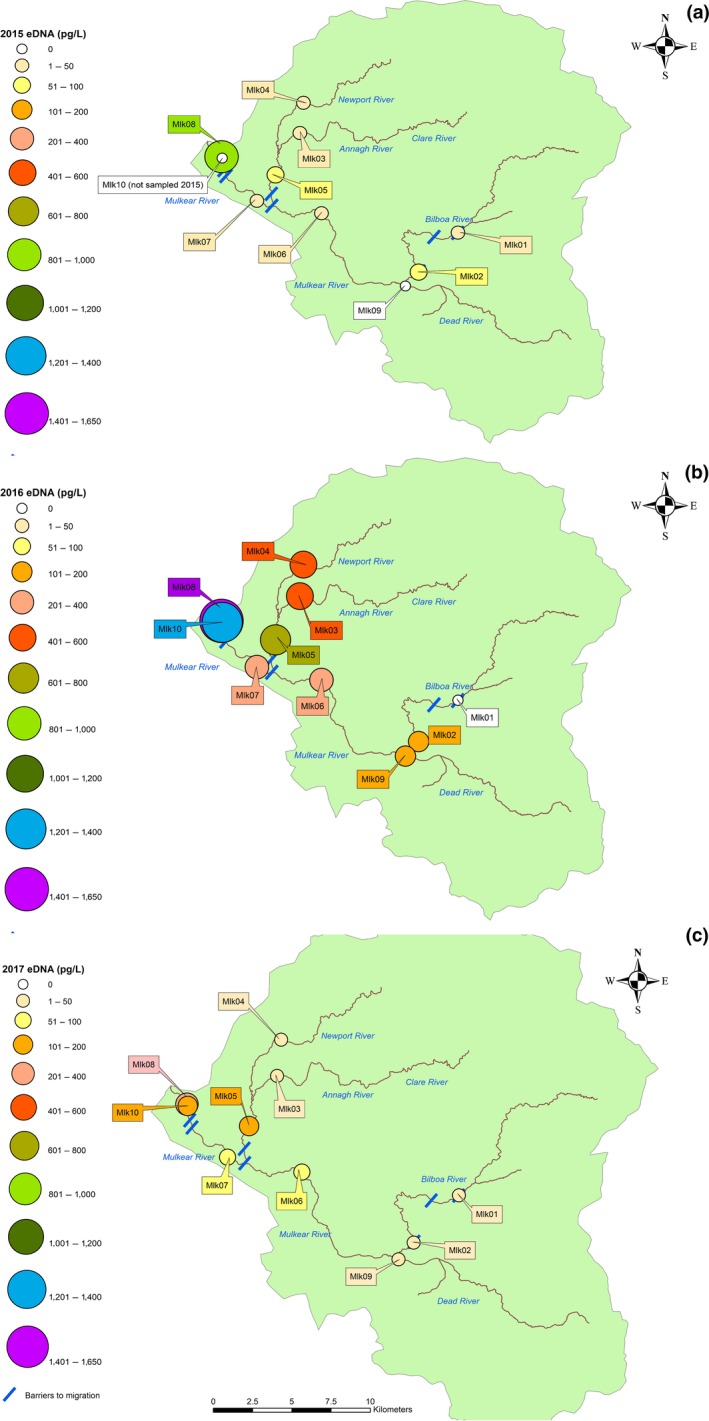
Total eDNA (pg/L) collected at each sampling site over the course of the spawning season in (a) 2015 (b) 2016 and (c) 2017 in the Mulkear catchment. Note that totals shown are cumulative concentrations at each sampling sites over each year and that number of sampling dates varies between years (See Figures 7 and 8)

Snapshot sampling was found to be successful in revealing spawning aggregations and habitat use within the Mulkear and MBW throughout the spawning season for all years sampled. All samples collected over each sampling year were combined (per site), which enabled the identification of relative habitat use and distribution within the catchment each year (Figures 3 and 4). Overall, eDNA concentration within the Mulkear catchment (with relatively higher sea lamprey densities and lower discharge) was noticeably higher than in the MBW catchment (with lower sea lamprey densities and higher discharge) as would be expected.

**Figure 5:**
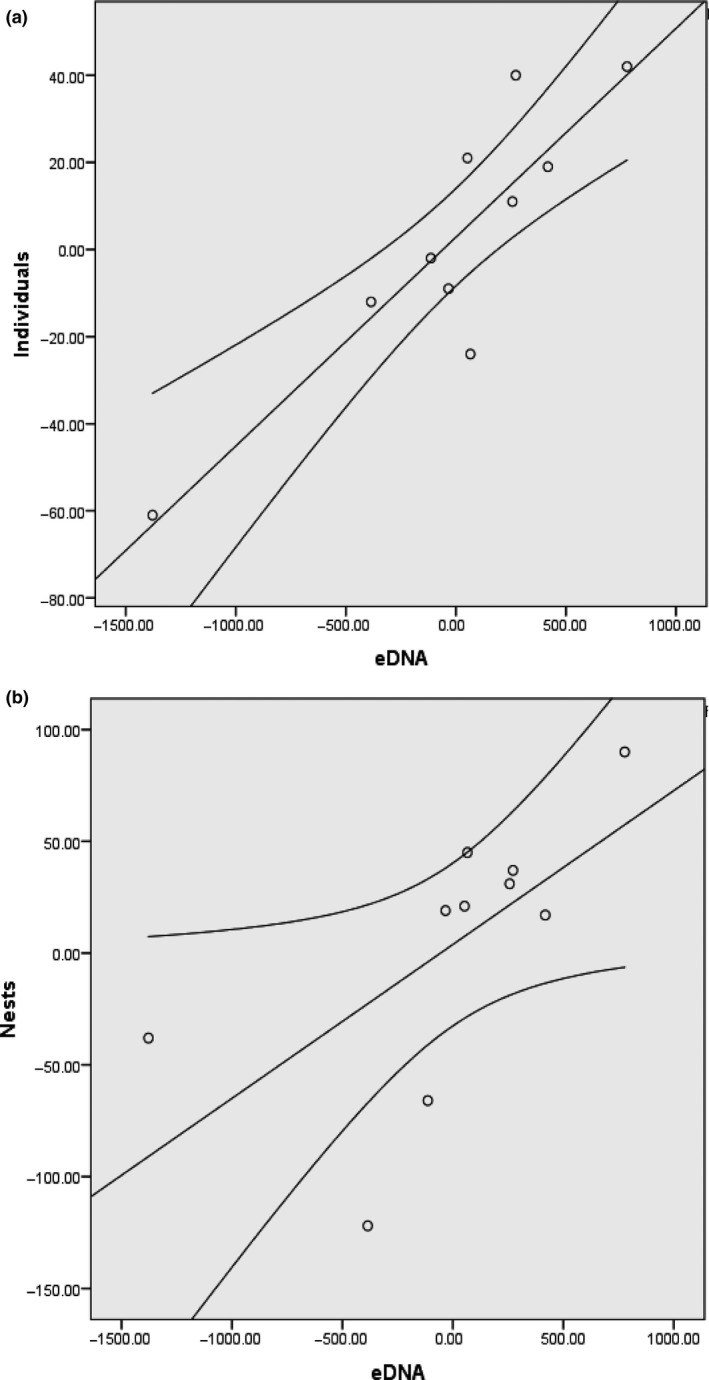
Significant positive correlation between eDNA concentration (pg/L) and (a) counts of individuals (Pearson's *R* = 0.886, *p* < 0.01) and (b) nest counts (Pearson's *R* = 0.644, *p* < 0.05) from below Annacotty weir (Mulkear) throughout the spawning season for years 2015–2017

**Figure 6:**
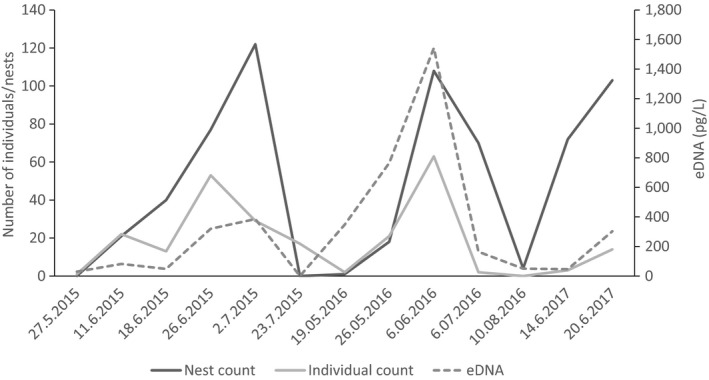
Graph showing the relationship between counts of nests/individuals and eDNA concentrations below Annacotty weir (Mulkear) throughout the spawning season for years 2015–2017. The eDNA concentrations used for 2017 are an average taken over the replicate samples

There was a significant positive correlation between the eDNA concentration (pg/L) and the number of individuals (Pearson's *R* = 0.886, *p* < 0.01) as well as with the number of nests (Pearson's *R* = 0.644, *p* < 0.05) counted below Annacotty weir (Figure 5). There is a clear relationship between eDNA concentration and both the number of individual lamprey and the number of nests counted at the sites below Annacotty weir (Figure 6). Unfortunately, no nest/individual counts could be carried out at Fermoy in the MBW due to severe turbidity and high water flows. Apart from the areas that are traditionally surveyed for lamprey spawning (i.e., Fermoy weir MBW09 and Annacotty MLK08 and MLK10), peaks in eDNA concentration were found in areas previously not identified as important habitat for *P. marinus *(e.g., MLK05, MLK06, MLK07). Snapshot sampling also showed the extent of the upstream distribution in both catchments (relative to sampling sites) revealing that in the MBW, *P. marinus *were able to reach as far as MBW06 which is over 100 km upstream from the mouth of the river, and in the Mulkear, *P. marinus *reached the uppermost sampling sites in all rivers sampled.

#### 3.1.1 Mulkear

Over the 3‐year sampling period, eDNA concentrations ranging from 0 to 831 pg/L were recorded in the Mulkear. All field controls and laboratory controls were found to be negative. In the Mulkear, *P. marinus *was detected in eight out of nine sites sampled in 2015, nine out of 10 sites in 2016, and all 10 sites in 2017 (Figure 4). Site MLK08 (most downstream site in catchment) generally exhibited the highest concentration of eDNA (Figure 4.). This was true for all years except one sampling date at the end of the spawning season in 2015 (2 July 2015), where MLK02 exhibited the highest relative concentration (41 pg/L). MLK05 also recorded relatively high eDNA concentrations, as did MLK02 in 2015; MLK04 and MLK03 in 2016; and MLK06 and MLK07 in 2017 (Figure 4). Of particular note was the detection of sea lamprey eDNA at MLK01, upstream of the gravel trap, a significant structure in terms of vertical height but one fitted with a Denil‐type fish pass. Wilcoxon signed‐rank tests revealed no significant difference between eDNA concentrations at each sampling site in the Mulkear catchment before (2015) and after (2016) the addition of the lamprey pass at Annacotty weir. This indicates that although the lamprey pass at Annacotty may assist lamprey in ascending the weir, there does not seem to be any significant difference in lamprey eDNA concentration at these sampling sites the year after the lamprey pass has been installed.

#### 3.1.2 Munster Blackwater

Over the 3‐year sampling period, eDNA concentrations ranging from 0 to 31.6 pg/L were recorded in the MBW which is overall much lower (nearly 27× lower) than that encountered in the Mulkear. Using eDNA, *P. marinus *were detected in two out of 11 sites in 2015, six out of 11 in 2016, and four out of 12 sites in 2017 (Figure 3). In 2017, site MBW10 had the highest relative concentration of eDNA/L (31.6 pg/L), followed in 2016 by Site MBW09 (23.4 pg/L) and in 2015 it was MBW11 (1.4 pg/L). In the MBW, MBW10 shows the highest concentrations of eDNA within this catchment; however, eDNA concentrations in the MBW showed generally lower variation and relatively smaller concentrations than the Mulkear. In both 2015 and 2016, eDNA was recorded at sites above Clondulane weir and Fermoy weir (Figure 2) showing that passage was possible at these locations.

### 3.2 “Snapshots” provide an overview of temporal distribution patterns and habitat use in the Mulkear

**Figure 7:**
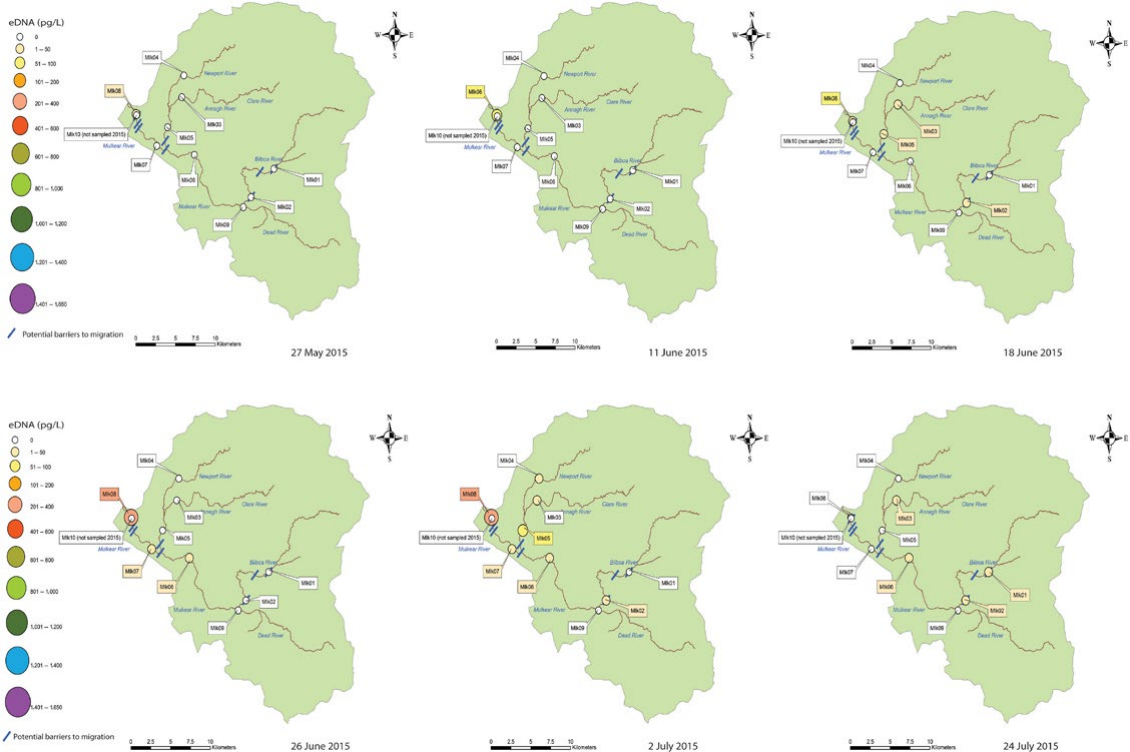
Snapshot sampling in the Mulkear in 2015 reveals temporal variation in eDNA concentrations taken over six separate sampling dates. Sampling on each date provides valuable information about dispersal throughout the catchment and habitat use over time

**Figure 8:**
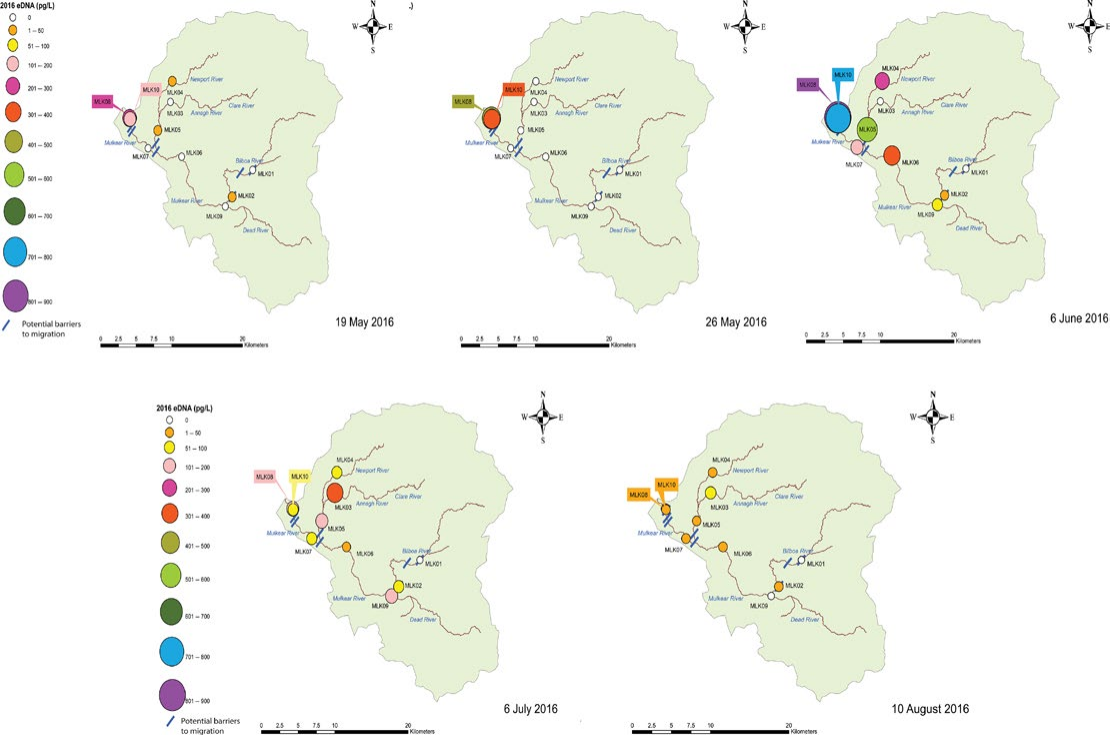
Snapshot sampling in the Mulkear in 2016 showing temporal variation in eDNA concentrations taken over five separate sampling dates. Sampling on each date provides valuable information about dispersal throughout the catchment and habitat use over time

Temporal variations in eDNA concentration throughout the Mulkear revealed fine‐scale patterns of movement over time (Figures 7 and 8). The overall pattern for both 2015 and 2016 showed eDNA initial detection and arise of eDNA concentration at MLK08 and MLK10 below Annacotty weir which commenced as soon as spawning activity began (i.e., visual confirmation of *P. marinus *and sampling commencing 27 May 2015 and 11 June 2016). In 2015, by June 18th, eDNA was detected at sites in the middle reaches of the Mulkear and the Newport Rivers and the concentration of eDNA rose at site MLK08 downstream of Annacotty. The final sampling in 2015 was on July 24th, when eDNA concentration was recorded at the highest point in the Bilboa river, but not at MLK08. *P. marinus *was not detected in the Dead River (MLK09) in 2015.

This was subsequently followed by upstream dispersal and eDNA detection at sites upstream of Annacotty weir. The eDNA detection at Annacotty continued to increase in concentration as the spawning season progressed (presumably as more *P. marinus *arrived at the sites and/or gametes were released into the system) until mid/late into the spawning season (the date of which varied year to year), at which time there is a visible drop in eDNA concentration at Annacotty coupled with a relative increase in eDNA concentration and detection at the sites further upstream (Figures 7 and 8). The eDNA snapshots from 2016 showed overall a higher relative concentration of eDNA and a difference in timescale from 2015—an earlier arrival and dispersal and an eDNA presence persisting into early August (i.e., a shift in the spawning season). Sampling on the 6 June 2016 showed the highest concentrations of eDNA recorded over the whole study (54% of the total DNA recorded in 2016) with 801–900 pg/L recorded at MLK08 (Figure 8), which corresponds to the highest number of nests counted at Annacotty in 2016 (Figures 5 and 6; 54% of all nests recorded in 2016 were counted on 6 June 2016). *P. marinus* eDNA was recorded at MLK09 on the Dead river on two occasions in 2016 (6th July and 6th June) but no eDNA was recorded at MLK01 in 2016. Similar pattern of occurrence was observed in two sampling dates in June 2017, with all sites showing positive eDNA for *P. marinus *except for MLK05. When comparing two replicate samples taken at each of the 10 sites, it was found that although there is some variation between replicate samples, the overall pattern of relative concentration remained the same between replicates. Correlation between samples was not significant on 14 June 2017, but on 20 June 2017 there was a significant positive relationship between the two samples (Pearson's *R = *0.990, *p* < 0.01).

## 4 DISCUSSION

The concentration of eDNA at any point in time is dependent on both the rate of production of eDNA (influenced by the level of activity of individuals, their metabolic rate, and behavior such as spawning, fighting etc.) as well as the density of the species within a system and the hydrology of the area. Therefore, the amount of eDNA in an environment will vary seasonally in response to environmental changes and the behavioral ecology of a given species (Barnes et al., 2014; Goldberg, Pilliod, Arkle, & Waits, 2011; Lacoursière‐Roussel et al., 2016). The increase in *P. marinus* biomass during the spawning season caused by the presence of large‐bodied adults, gametes, and later their carcasses in the river system greatly increased the chance of detecting our target species and created an ideal opportunity to monitor patterns of spatio‐temporal distribution and habitat use within two important SACs in Ireland. Overall, we found eDNA concentration and detection during the spawning season were noticeably higher in the Mulkear catchment than in the MBW catchment which might be expected considering the relative densities of the target species and the differing discharge volumes within each catchment. Due to positive results from the *S. trutta *assay with all the MBW and MLK samples, we can rule out false negatives due to the presence of inhibitory substances within the samples that may cause PCR issues. This reinforces the usefulness and sensitivity of the assay to detect species in fast flowing, turbid water even at relatively low abundances. Compared to 2016 and 2017, the samples taken in 2015 have generally lower eDNA concentrations at each sampling site. However, as we can see from the results above, there is very little difference in the number of nests/individual lamprey encountered at Annacotty between 2015 and 2016 so presumably there was no great difference in the number of lamprey present in the catchment. The difference in eDNA concentration from 2015 and 2016 may consequently be due to the difference in storage methods used (i.e., 2015 samples were stored in ethanol and samples were frozen as in 2016 and 2017). Previous studies have shown a lower eDNA yield from filters preserved in ethanol as compared to other storage methods (Majaneva et al., 2018).

In the Mulkear, site MLK08 (most downstream site in catchment) generally exhibited the highest concentration of eDNA as would be expected due to its location within the largest known spawning site for *P. marinus* in Ireland (incorporating also MLK10). We observed a positive correlation between spawning activity (measured as number of individuals/nests) and eDNA concentration, which is consistent with other studies (Doi et al., 2017; Pilliod et al., 2013; Takahara et al., 2012; Thomsen et al., 2012). Bylemans et al. (2017) have previously shown that the spawning events of the Macquarie perch (*Macquaria australasica*) were also characterized by higher concentrations of eDNA. However, interactions between distance and flow may be confounding factors in attempts to infer abundance at a location based on eDNA sampling in running water. Where eDNA was positively detected at our sites, we cannot rule out the possibility that eDNA was drifting downstream from another area which either compounded the concentrations we detected or did not geographically represent the exact location of critical spawning habitat. In the Mulkear catchment, however, sampling sites with positive detection were found to be downstream of areas where nests have been encountered in previous years and can therefore be validated. The MBW sites, however, were not connected to nest sites in previous years (except for MBW09 and MBW 10).

Strong temporal increases in eDNA during months associated with breeding have also been observed in a number of amphibians such as the eastern hellbender (*Cryptobranchus alleganiensis alleganiensis*), and Chinese and Japanese giant salamanders (*Andrias davidianus *and *A. japonicus *respectively) (Buxton, Groombridge, Zakaria, & Griffiths, 2017; Fukumoto, Ushimaru, & Minamoto, 2015; Spear, Groves, Williams, & Waits, 2015). Spawning lampreys are traditionally monitored using walkover surveys and can be quite visible in some systems, however, they are far more difficult to observe in larger or more turbid river systems (Johnson, Buchinger, & Li, 2015). During periods when walkover surveys were not possible due to turbidity, in this study eDNA sampling was still possible at these times. Therefore, eDNA sampling allowed effective monitoring during periods, when surveyors dependent on traditional survey techniques alone may have been unable to collect data.

Generally, the choice of sampling and extraction methods for eDNA studies are dependent on cost, sampling location, preference, and species/ecosystem consideration. Evans, Shirey, Wieringa, Mahon, and Lamberti (2017) detected brook trout (*Salvelinus fontinalis*) via both electrofishing and eDNA. The eDNA analysis required lower sampling effort and cost 67% less than triple‐pass electrofishing. However, eDNA was more expensive than presence–absence electrofishing, and currently, no information regarding population structure can be obtained from eDNA sampling. Our sampling strategy coupled with the low‐cost extraction method using Chelex makes our approach accessible to conservation and fisheries managers. Potential per sample costs for eDNA extraction with Qiagen's DNeasy^®^ Blood and Tissue kit are ~€3.80–€4.30 per sample as compared to the Chelex extraction protocol are ~€0.01–€0.05 per sample. eDNA can consequently be utilized as an invaluable tool to complement traditional survey techniques and fill in knowledge gaps where these methods may not be comprehensive but traditional surveys still contribute valuable information to conservation managers.

Snapshot sampling also allowed the identification of peaks in eDNA concentration in areas that were not previously identified as important habitat for *P. marinus *in Ireland (e.g., MLK05, MLK06, MLK07 and then MLK03 and MLK09 later in the season). Although these peaks in eDNA concentration are indicative of increased *P. marinus* densities within these areas, without having more information about eDNA degradation rates, and flow rates within these areas, it cannot be ascertained how much eDNA is dispersed from areas upstream of these sites. However, this does outline areas of interest for future spawning surveys. This study has shown that eDNA snapshot sampling can be effectively used to identify areas of critical habitat for low‐density species of conservation concern such as *P. marinus*. The results above have shown that eDNA can be very effective in outlining the general locations of spawning aggregations as well as the upstream extent of migrating individuals relative to potential migration barriers within a catchment. Not only can specific areas be identified for the focus of future spawning surveys, but the magnitude of a spawning aggregation relative to other sites, or other years, can also be very useful for future management decisions.

Currently, literature dealing with running waters is still ambiguous about the effect on the downstream transportation of DNA (Roussel et al., 2015). In the study conducted by Gingera et al. (2016), water samples that were taken 1–2 km downstream had higher detection frequencies (75%–80%) than those collected at the most upstream site (approximately 50%). This suggests that, for a general management application, the chances of detecting a target species is increased if sampling is performed lower in the watershed, presumably because downstream sampling integrates the eDNA from a larger number of the target organism. Laramie, Pilliod, and Goldberg (2015) quantified the eDNA of Chinook salmon (*Oncorhynchus tshawytscha*) in relation to stream location and found no consistent relationship between stream distance and eDNA concentration. This would indicate that eDNA is not accumulating in downstream reaches, but is instead being removed through processes such as settling or destruction from physical forces (Piggott, 2016). This hypothesis is further supported by the work of Jane et al. (2015), who found that the distance eDNA travelled from the source was reduced at low flows due to a combination of cell settling, turbulence, and dilution effects. Nonetheless, our results have shown that, without sampling at many locations throughout a catchment, fine‐scale patterns of movement and habitat use may be overlooked. Arguably, these are some of the most prevalent concerns when considering the best management practices for a conservation species.

The potential power of eDNA as a conservation tool is not fully exploited when only presence and absence are considered. This study has also highlighted that without a prior knowledge of the biology and ecology of a target species, it would be extremely difficult to ascertain if increased eDNA concentration at a point in time reflects higher densities of the target organism, or if there is a behavioral/environmental reason for an increase in eDNA concentration/detectability. Therefore, prior knowledge of target species’ biology/ecology is crucial in the interpretation of the results of future eDNA studies. A better understanding of the way in which eDNA disperses and persists in a system will also greatly improve future sampling design and maximize the likelihood of detection. However, we have here shown that utilizing knowledge about the ecology of a target species can greatly improve not only the chances of detection, but also improves the complexity of the information discernible from the eDNA samples.

## CONFLICT OF INTEREST

None declared.

## AUTHOR CONTRIBUTION

F.B., J.C., J.K., and M.K.‐Q. came up with concept for the study. FB and SR conducted all fieldwork. F.B. and J.C. carried out lab work and analysis. All authors discussed the results and implications. F.B. wrote the manuscript and all authors contributed to the final manuscript.

## DATA ACCESSIBILITY

Sampling locations and Data are archived in DRYAD https://doi.org/10.5061/dryad.j6h56qg.
